# High levels of unbound bilirubin are associated with acute bilirubin encephalopathy in post-exchange transfusion neonates

**DOI:** 10.1186/s13052-021-01143-z

**Published:** 2021-09-15

**Authors:** Yiyi Ding, Shuo Wang, Rui Guo, Aizhen Zhang, Yufang Zhu

**Affiliations:** 1 Department of Pediatrics, The First People’s Hospital of Changde, Changde, 415003 China; 2 Jishou University School of Medicine, Jishou, 416007 China

**Keywords:** Neonatal, Acute bilirubin encephalopathy, Exchange transfusion, Unbound bilirubin

## Abstract

**Background:**

Although it is known that unbound bilirubin can enter the brain, there is little evidence of its association with the development of acute bilirubin encephalopathy. Here, we investigated this potential relationship in neonates who had undergone exchange transfusion.

**Methods:**

Data from 46 newborns who underwent exchange transfusion between 2016 and 1-1 to 2018-12-31 at the First People’s Hospital of Changde City in China were analyzed. The unbound bilirubin level was taken as the independent variable and the development of the acute bilirubin encephalopathy as the dependent variable. The covariates were age, birth weight, sex, red blood cell count, blood glucose, hemolytic disease, and whether the infant had received phototherapy.

**Results:**

The mean age and gestational age of the neonates were 146.5 ± 86.9 h and 38.6 ± 1.3 weeks [38.7(34.6–41.1) weeks] old, respectively; 52.17% were male. Binary logistic regression analysis after adjustment for covariates showed a positive association between the levels of unbound bilirubin and the development of acute bilirubin encephalopathy (odds ratio = 1.41, 95% confidence intervals 1.05–1.91, *P* = < 0.05).

**Conclusion:**

There is a significant association between unbound bilirubin levels and the development of acute bilirubin encephalopathy in neonates. Further investigations are required to explore the mechanisms.

## Background

Neonatal jaundice is common as the result of elevated bilirubin levels which may be unbound bilirubin (UB) and/or conjugated bilirubin [1]. Severe neonatal hyperbilirubinemia (SNH) occurs when the total serum bilirubin (TSB) at the exchange transfusion (ET) threshold as a function of postnatal age and gestational age is ≥20 mg/dL or 342 μmol/L, or if elevated bilirubin is combined with the symptoms of acute bilirubin encephalopathy (ABE) [2–4]. Bilirubin levels often increase soon after birth and generally follow their hour-specific percentile track, remaining within their risk zones and declining by the end of the first week [1]. However, neonatal jaundice still occurs in 60–80% of newborns, while SNH may result in serious long-term neurodevelopmental consequences and even death, particularly in low to middle income countries [2, 5–7]. A systematic review reported geographical differences in the overall incidence of SNH globally differently, which was highest in Africa (667.8/10000), followed by southeast Asia (251.3/10000), the eastern Mediterranean (165.7/10000) and western Pacific region (9.4/10000). The Americas and Europe both had substantially lower incidences of 4.4 and 3.2 per 10,000 live births, respectively [8]. As many as 24 million newborns may suffer adverse outcomes. For example, the incidence of kernicterus with Rh disease ranges from 25 to 38 per 100,000 live births in various European, Asian, African, and Latin American countries, leaving most survivors impaired [7]. One study identified 47 infants with TSB > 30 mg/dL (8.6 per 100,000 births), of which 8.5% exhibited ABE [9].

The symptoms of ABE include irritability, lethargy, reduced muscle tone, apnea, and convulsions [1, 10–12]. Phototherapy and ET are commonly used to avoid the development of kernicterus and other consequences. ET is recommended if the TSB rises to levels above the recommended age- and gestational age-specific TSB thresholds, or in the presence of symptoms indicative of ABE despite intensive phototherapy [3, 12, 13]. The efficacy of ET in reducing the TSB has been demonstrated [14]. However, Wusthoff et al. have argued that, in the absence of a linear relationship between the TSB and the risk of bilirubin-induced neurologic dysfunction (BIND), other hyperbilirubinemia measures, such as UB or the bilirubin-albumin binding capacity, may be more relevant [15]. Acidosis, dehydration, sepsis, rate of serum bilirubin increase, and hypoalbuminemia are regarded as risk factors for ABE [1, 12]. The UB level can also be used to determine the risk of hyperbilirubinemia. Chronic high UB levels may also indicate a risk of kernicterus in low birth-weight neonates [16]. The potential usefulness of UB in evaluating BIND has been documented [17].

Non-water soluble UB is converted in the hepatocyte to the water-soluble conjugated by the enzyme uridine-di-phospho-glucuronosyl-transferase and is excreted into the gut where some of the conjugated bilirubin is reconverted to UB and resorbed into the enterohepatic circulation [1]. As UB can pass through the blood-brain barrier, the plasma-free bilirubin level may be preferable for assessing the risks of neurological damage than TSB [18–20]. UB may be deposited in the basal ganglia, the auditory passage, and the movable core muscle. This deposition and its accompanying damage lead to the typical symptoms of kernicterus. In premature infants, the damage threshold of bilirubin is approximately 14 mg/dL with increased risk of damage, as the UB serum levels rise [19]. Certain factors, including preterm birth, hypoxia, acidosis, seizures, hypoalbuminemia, and sepsis, are thought to increase the risk of ABE; these factors may also promote the translocation of bilirubin into the brain [12] (Fig. 2).

There is, however, limited information on the relationship between UB levels and ABE, especially in cases of SNH. As ET is used in cases where the bilirubin has reached a certain level, we aimed to investigate whether UB levels are independently related to ABE in neonates undergoing ET.

## Materials and methods

The data were stored in the hospital’s electronic database and were anonymous to protect the participants’ privacy. The study was approved by the Ethics Committee of our institution and complied with the Code of Ethics of the Declaration of Helsinki. The independent variable in this study was the UB level measured before ET. The dependent variable was the occurrence of ABE. The study population comprised neonates admitted to the Neonatology Department of the First People’s Hospital of Changde from January 2016 to December 2018 with the inclusion criteria being infants hospitalized in the Neonatology Department of our hospital, diagnosed with neonatal hyperbilirubinemia, and having undergone an ET.

ETs were always performed with written informed parental consent. Detailed explanations of the benefits and risks of the procedure and the potential consequences of not performing the procedure in infants with SNH were provided. Finally, 75 patients met the ET criteria [3]. Of these, 29 patients’ parents refused consent to perform ET; these included one patient with ABE. ETs were performed only by trained personnel in our neonatal intensive care unit with full monitoring and resuscitation capabilities. Phototherapy for all the 75 patients was continued. On diagnosis of neonatal hemolytic disease, the patient received intravenous immunoglobulin 0.5–1 g/kg over 2 h, repeated in 12 h if necessary.

Baseline UB and TSB levels were obtained before ET using the Beckman Coulter Chemistry Analyzer AU5800 and the Vanadate Oxidation method. We then extracted data for each patient including age, birth weight, blood glucose, white blood cell count, weight, and sex, as well as the presence of an ABE diagnosis, hemorrhage, hemolysis, and infection (Table 1). Specifically, hemorrhage included scalp hematoma, and abdominal and intracranial hemorrhage detected by ultrasonography and magnetic resonance imaging, respectively. Hemolysis included hemolytic disease of the newborn as a result of ABO and RhD incompatibility (the diagnosis required a documented reticulocytosis and/or positive Coombs test), as well as autoimmune hemolytic disease and glucose-6-phosphate dehydrogenase (G6PD) deficiency, while infection included pulmonary and intracranial infection, as well as omphalitis and sepsis.

**Table 1:** Baseline characteristics of participants (*n* = 46)

ABE	No (*n* = 28)	Yes (*n* = 18)	*P*-value
**Demographic data**			
Age, mean (SD), hours	128.7 (94.2)	174.2 (67.6)	0.042
Gestational age, mean (SD), weeks	38.8 (1.5)	38.3 (0.9)	0.205
Birth weight, mean (SD), kilogram	3.28 (0.35)	3.25 (0.45)	0.825
Weight, mean (SD), kilogram	3.14 (0.39)	3.13 (0.41)	0.923
Male, No. (%)	13 (46.43%)	11 (61.11%)	0.331
**Laboratory data**			
TSB_1_, mean (SD), mg/dL	25.96 (5.53)	33.77 (5.94)	< 0.001
UB, mean (SD), mg/dL	23.74 (5.12)	30.35 (5.54)	< 0.001
TSB_2_, mean (SD), mg/dL	11.13 (3.32)	17.53 (6.27)	< 0.001
Blood glucose, mean (SD), mmol/L	5.3 (1.2)	6.9 (3.0)	0.017
White blood cell, mean (SD), *10^⋀^9/L	13.2 (5.1)	14.2 (4.7)	0.508
Blood platelet count, mean (SD), *10^⋀^9/L	289.7 (125.6)	264.0 (116.0)	0.493
Red blood cell, mean (SD), *10^⋀^12/L	4.07 (1.08)	3.29 (0.72)	0.010
Hemoglobin, mean (SD), g/L	143.2 (35.5)	116.1 (29.6)	0.011
Serum sodium, mean (SD), mmol/L	142.5 (3.0)	142.1 (2.6)	0.584
Serum calcium, mean (SD), mmol/L	2.40 (0.25)	2.38 (0.17)	0.762
Serum potassium, mean (SD), mmol/L	4.62 (0.52)	4.39 (0.38)	0.117
Serum albumin, mean (SD), g/L	37.45 (2.80)	36.12 (4.16)	0.201
Acidosis, No. (%)	5 (17.86%)	6 (33.33%)	0.230
Hypoxia, No. (%)	1 (3.57%)	2 (11.11%)	0.552
**Cause of severe hyperbilirubinemia**			
Hemorrhage, No. (%)	6 (21.43%)	3 (16.67%)	0.691
Hemolysis, No. (%)	16 (57.14%)	12 (66.67%)	0.518
Infection, No. (%)	11 (39.29%)	8 (44.44%)	0.729
Erythrocytosis, No. (%)	2 (7.14%)	0 (0.00%)	0.246
Unknown reason, No. (%)	1 (3.57%)	3 (16.67%)	0.124
**Treatment**			
Iv immunoglobulins, No. (%)	6 (21.43%)	7 (38.89%)	0.199
Receive phototherapy before ET, No. (%)	25 (89.29%)	11 (61.11%)	0.024

TSB_1_:TSB at the start of ET

TSB_2_:TSB at the end of the ET

Hemorrhage: include scalp hematoma (*n* = 2), subarachnoid hemorrhage (*n* = 2), subdural hemorrhage(*n* = 1), and intracranial hemorrhage(*n* = 4) showed by MRI

Hemolysis: include ABO haemolytic, RhD haemolytic, Autoimmune haemolytic and Glucose-6-phosphate Dehydrogenase defificiency

Infection: include sepsis, pulmonary infection, intracranial infection and umbilical infection

### Statistical analysis

Means and standard deviations were used to express normally distributed continuous variables and medians (min, max) for non-normal distributions. Categorical variables were expressed as frequencies or percentages. Student’s t-test was used to analyze differences between continuous variables and the χ2 test was used for categorical variables. The relationship between UB and ABE was analyzed by multivariate logistic regression and three models were constructed to illustrate the stability of this relationship: Model 1, no adjustment for covariates; Model 2, adjustment only for sex, age, and birthweight; and Model 3, which used model 2 together with the other covariates listed in Table 2. Since blood glucose levels, red blood cell count, and phototherapy administration before ET were statistically different between the two ABE groups (Table 1), multivariate regression analysis adjusting for the effects of these covariates on ABE was necessary. As several reports have suggested that hemolytic disease is associated with ABE or ET [11, 21–23], we adjusted for these covariates in Table 2. All analyses were performed with the statistical software packages in R (http://www.R-project.org, The R Foundation) and EmpowerStats (http://www.empowerstats.com, X&Y Solutions, Inc., Boston, MA). *P*-values <0.05 (two-sided) were considered statistically significant.

**Table 2:** Relationship between UB VS ABE in different models

	OR(95%CI)	*P*-value
Non-adjusted	1.29 (1.10, 1.52)	0.002
Adjust I	1.34 (1.11, 1.63)	0.003
Adjust II	1.41 (1.05, 1.91)	0.025

Result variable: ABE

Exposure variable: UB

Non-adjusted model adjust for: None

Adjust I model adjust for: Sex; Age; Birth weight;

Adjust II model adjust for: Sex; Age; Birth weight; Blood glucose; Red blood cell; Hemolysis; Receive phototherapy before ET;

## Results

### Baseline characteristics of participants

Over the three-year period, 5317 neonates were admitted. Of these, 1776 late preterm and term infants (33.4%) were diagnosed with neonatal hyperbilirubinemia defined by the percentile on the nomogram, as recommended by the American Academy of Pediatrics (AAP). Phototherapy and ET were performed as per the AAP guidelines [3]. Standard and specialized treatment was given to every patient according to the cause of the SNH after the initial investigations. Thirty-six (78.3%) of the infants had received multiple phototherapy sessions before deciding and preparation for the ET, with 13 (28.3%) receiving intravenous immunoglobulins. Of these 46 neonatal ET cases, 12 (26.1%) were from the maternity unit, 26 (56.5%) from home and 8 (17.4%) neonates were transferred from other hospitals. Phototherapy for all 46 patients was continued during the preparation for ET and during and after the procedure itself. All treatments were performed according to the NICE Neonatal Jaundice Clinical Guidelines 2010(published 19 May 2010, last updated 26 October 2016) [12].

**Fig. 1:**
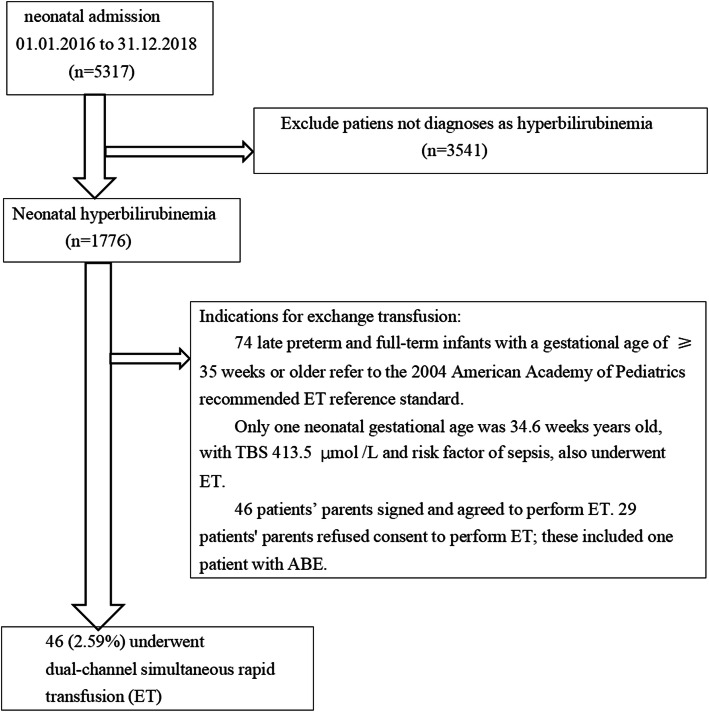
Inclusion and exclusion criteria

Screening according to the inclusion and exclusion criteria resulted in the selection of 46 participants for the final data analysis (see Fig. 1 for flowchart). Table 1 shows the participant characteristics in terms of their ABE diagnoses. The average age of the 46 participants was 146.5 ± 86.9 h and 52.17% were male. All 46 infants received cranial magnetic resonance imaging. Nine infants were diagnosed with hemorrhage (19.6%), including scalp hematoma (*n* = 2), subarachnoid hemorrhage (*n* = 2), subdural hemorrhage (*n* = 1), and intracranial hemorrhage (*n* = 4). Twenty-eight infants were diagnosed with hemolysis (60.9%), including ABO (*n* = 8), RhD (*n* = 2), and autoimmune hemolytic disease (*n* = 2). Sixteen infants had G6PD and 19 were diagnosed with infection (41.3%). No significant differences were observed in gestational age, birth weight, white blood cell count, sex, hemorrhage, hemolysis, or infection between the ABE and non-ABE groups (all *P*-values > 0.05). There were significant differences in age, blood glucose, red blood cell count, hemoglobin, TSB_1_ (TSB at the start of ET), UB, TSB_2_ (TSB at the end of ET), and phototherapy administration before ET between the ABE and non-ABE groups (*P* < 0.05) (Table 1).

**Fig. 2:**
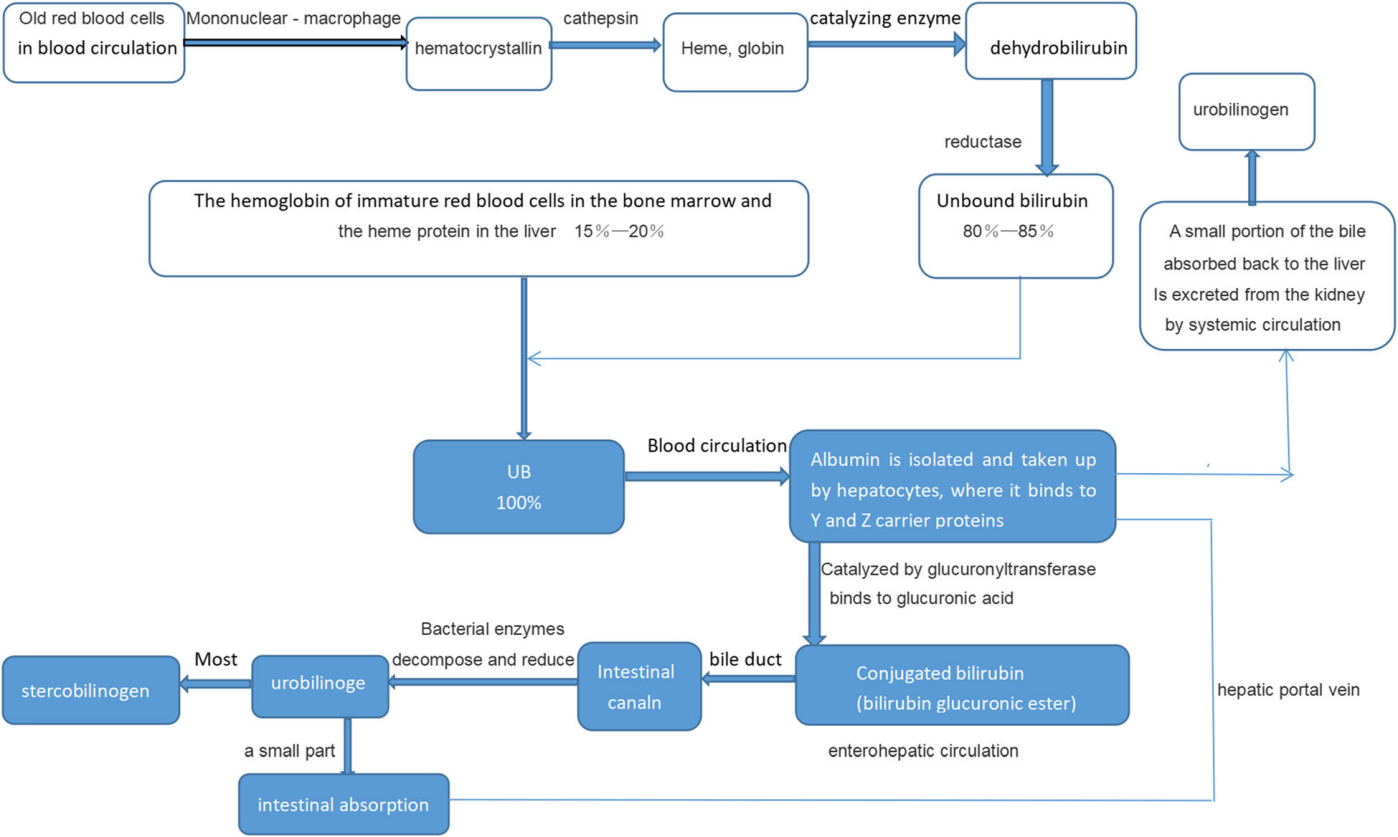
Bilirubin metabolic cycle

### Adjusted and non-adjusted binary logistic regression

The independent effects of UB levels on ABE were analyzed in three models using multivariate binary logistic regression. The effect sizes (odds ratios) and 95% confidence intervals are listed in Table 2. In the non-adjusted Model 1, the UB level (1 mg/dl) accounted for ABE risk. In Model 2 (adjusted for sex, age, and birth weight), the odds ratio (OR) was 1.34, 95% with confidence intervals (CI) of 1.11 to 1.63 (P < 0.05). In Model 3 (adjusted for all covariates shown in Table 2, including sex, age, birth weight, blood glucose, red blood cell count, hemolysis, and administration of phototherapy before ET), the effect size (OR:1.41, 95% CI 1.05, 1.91) was also stable.

### Follow-up for 2 years after discharge

Of these 46 cases, 3 died within 1 week of discharge, 14 were lost to follow-up, and 22 were followed up without abnormalities. There were 4 of weight loss, 1 of dwarfism, 3 of abnormal motor development, 6 of abnormal language and social development, 2 of abnormal cognitive development, 4 with hearing disorders, 4 of abnormal dental development, and 2 cases of tardive dyskinesia, twitching, or other limb movement disorders.

## Discussion

Bhutani et al. calculated the chronic kernicterus risk as one in seven for infants with TSB > 30 mg/dL [24]. One study suggested that TSB (above 25 mg/dL) was a poor predictor of ABE when other risk factors were present [25]. Another study reported that raised UB levels are associated with higher mortality or poor neurodevelopmental outcomes despite any clinical condition. Increased TSB levels are directly related to an increased risk of unstable, as opposed to stable, infant death and neurodysplasia [26]. UB is still one of the main reasons for neonatal morbidity and hospitalization. Occasionally, UB can reach critically high levels and cause brain injury [27]. The measurement of UB is important for assessing the risk of neurotoxicity and for proper intervention in high-risk neonates with hyperbilirubinemia [28].

Lower UB concentrations can cause neuronal apoptosis, while high concentrations of UB can lead to necrosis. Bilirubin toxicity is more likely to affect the brain stem nuclei and basal ganglia with neurotoxicity resulting from excess glutamate production, mitochondrial dysfunction, the action of pro-inflammatory cytokines, and raised intracellular calcium levels [29, 30]. Moderate to high UB levels are associated with oxidative stress and the changes caused by oxidative stress may be early predictors of adverse outcomes. Lower bilirubin levels can also cause DNA damage, suggesting that UB may have genotoxic effects [27]. Experimental studies on brain tissue exposed to UB have shown axonal damage, including significant decreases in myelination, fewer compact axons, and the presence of debris [31]. The presence of UB in the brain can cause neurological dysfunction, the acute form of which is ABE.

In this study, 46 infants received ET; the mean UB value in these children was 450.1 ± 105.4 μmol/L and 18 were diagnosed with ABE. Patients who met the ET criteria as per AAP guidelines [3] but did not receive ET treatment were not included in the analysis. Twenty-nine patients’ parents refused consent to perform ET; this included one patient with ABE. There were significant differences in the TSB values between the 29 infants (24.46 ± 2.80 mg/dL) who did not receive ET and the 46 infants (29.02 ± 6.82 mg/dL) who received ET. The TSB levels in most of the 29 infants were not far above the ET threshold level, and there was no nervous system involvement. Neither the doctors nor the families had a positive attitude towards ET. All the 29 infants received phototherapy and were fully recovered on discharge. It is possible that parental consent may have been influenced by the pediatricians descriptions of the severity of the disease. While we cannot completely rule out some influence on the results using these data, the data used in the analysis include almost all of the ABE patients, which we consider meaningful for discussing the relationships of UB levels with ABE in SNE.

Ebbesen et al. [32] identified 32 infants in whom the TSB values exceeded the indications for ET, 11 with evidence of ABE. The exact level of bilirubin that is likely to cause neurotoxicity in any individual baby varies, and depends on the interplay of multiple factors. Correlations between ABE and circulating bilirubin levels are poor [12]. Brito et al. reporting on a preterm neonate with kernicterus, proposed that UB increases the blood vessel density in the hippocampus and striatum related to the nucleus macula, triggering an immune response mediated by VEGF and VEGFR-2, and allowing albumin infiltration into the brain [33]. When the bilirubin-binding ability of the blood is raised or when there is competition for the bilirubin-binding site on albumin, UB enters the cerebrum. Gestational age, hemolysis, infection, sepsis, and, particularly, Rh isoimmunization, which are associated with neuronal susceptibility, are additional risk factors for kernicterus [11]. Measures to prevent extreme jaundice and reverse neurotoxicity include measuring bilirubin levels during the infant’s stay in the maternity ward, assessing other risk factors (such as the presence of possible hemolytic diseases, hypothermia, hypoglycemia, and sepsis, amongst others), and educating parents. Emergency treatment should include immediate phototherapy, consideration of intravenous immunoglobulin administration, and preparation for ET treatment [2, 10, 12]. In our data, we found no significant difference in infection, hemolysis, and gestational age between the ABE and non-ABE groups, which may be a consequence of the relatively small sample size (Table 1).

Our research included 5317 neonatal admissions over 3 years, of which 1776 (33.4%) were admitted for neonatal hyperbilirubinemia. The incidence of ABE and ET were 10.7% (*n* = 19) (including 3 deaths) and 25.9% (*n* = 46), respectively. Similarly, one study reported that the incidence of bilirubin encephalopathy in hyperbilirubinemic infants ranged from 7 to 22% between centers [21]. Another study of 1118 hyperbilirubinemic infants reported that the incidence of ABE and ET were 17.0 and 31.5% respectively [23]. This study also identified the peak TSB level as predictive of ABE, while peak TSB, ABE, and ABO incompatibility were predictors of ET [23]. The risk evaluation of kernicterus based on TSB levels alone has often proved insufficient; TSB levels at the start of ET also lack uniformity even in the presence of the clinical symptoms of ABE and hemolytic disease. Although ET is an effective treatment for BIND in newborns with SNH, it nevertheless carries risks and should be used only after a careful assessment of the risk of kernicterus [4]. The precise role of TSB and UB in calculating ABE risk remains unknown. However, data, both clinical and laboratory, indicate that measurement of UB is superior to TSB in detecting the bilirubin toxicity risk in SNH [34].

Emerging evidence suggests that UB may be superior to TSB in predicting BIND in both pre-term and term infants [28, 35, 36]. Our study considered the effects of age, sex, red blood cell count, birth weight, blood glucose, hemolysis, and phototherapy administration before ET on the UB levels and ABE relationships when adjusting for covariates (OR: 1.41, 95%CI 1.05, 1.91) (Table 2). This finding is consistent with several reports showing a significant relationship between UB, rather than TSB or the bilirubin: albumin ratio and chronic auditory toxicity [17, 37]. Another report showed a significant association between auditory brainstem damage and UB, but not TSB, concentration [38]. Similarly, one study showed that UB levels were significantly associated with kernicterus development [39].

In our study, we observed significant differences in age, blood glucose, red blood cell count, UB, and phototherapy administration before ET between the ABE group and non-ABE groups (*P* < 0.05) with the occurrence of ABE. The results were maintained in the fully adjusted model (Model 3) where all covariates shown in Table 2 were adjusted for each additional mg/dL of the UB levels.

The present study offers the first documentation of an independent association between UB levels and ABE in infants who had undergone ET. These findings should assist future research on the diagnosis and prediction of ABE. Our study has several limitations. Firstly, as the research subjects were infants who had undergone ET, the findings may lack universality and, secondly, because ABE neonates who had not undergone ET were excluded, the findings may be further restricted in their application.

## Conclusion

UB levels are independently associated with neonatal ABE in children who underwent ET. The physiological mechanism underlying the relationship between UB levels and neonatal ABE requires further exploration.

## Data Availability

The datasets used and analysed during the current study are available from the corresponding author on reasonable request.

## Abbreviations

UB: Unbound bilirubin
SNH: Severe neonatal hyperbilirubinaemia
TSB: Total serum bilirubin
ET: Exchange transfusion
ABE: Acute bilirubin encephalopathy
BIND: Bilirubin-induced neurologic dysfunction
G6PD: Glucose-6-phosphate dehydrogenase
AAP: American Academy of Pediatrics
OR: Odds ratio
CI: Confidence intervals
