# Correction: High levels of unbound bilirubin are associated with acute bilirubin encephalopathy in post-exchange transfusion neonates

**DOI:** 10.1186/s13052-023-01432-9

**Published:** 2023-02-15

**Authors:** Yiyi Ding, Shuo Wang, Rui Guo, Aizhen Zhang, Yufang Zhu

**Affiliations:** 1 Department of Pediatrics, The First People’s Hospital of Changde, Changde, 415003 China; 2 Jishou University School of Medicine, Jishou, 416007 China

**Correction: Italian Journal of Pediatrics 47, 187 (2021)**

**https://doi.org/10.1186/s13052-021-01143-z**

After publication of our article [1] we became aware that throughout the article, and including the title, the term “unbound bilirubin” should be replaced by “indirect (unconjugated) bilirubin”.
